# The relationship between teacher–student relationships and academic grades among Chinese rural high school students: the moderating role of mental health symptoms and the conditional moderating effect of academic resilience

**DOI:** 10.3389/fpsyg.2024.1416783

**Published:** 2024-10-30

**Authors:** Xiaohui Chen, Richard Peter Bailey, Xiaojiao Yin, Nadia Samsudin

**Affiliations:** ^1^Faculty of Social Sciences and Liberal Arts, UCSI University, Kuala Lumpur, Malaysia; ^2^UCSI University, Kuala Lumpur, Malaysia

**Keywords:** educational psychology, psychosocial factors in learning, quality education, education environment, mental well-being

## Abstract

**Objective:**

This study examines the relationship between Teacher–Student Relationships and academic grades among Chinese rural high school students, focusing on the moderating role of mental health symptoms and the conditional moderating effect of academic resilience.

**Method:**

A moderated moderation analysis was conducted via Mplus on data collected from a sample of rural Chinese high school students. SEM was used to test the direct and interactive effects of these variables on academic outcomes.

**Results:**

Teacher–Student Relationships were found to have a significant positive association with students’ academic grades. Academic resilience plays a conditional moderating role, with students who have higher levels of resilience better able to maintain their academic performance, even when facing psychological distress. This suggests that resilience can buffer the impact of challenges, enhancing the positive influence of TSRs on academic outcomes.

## 1 Introduction

In high school education, academic grades serve as more than just markers of learning; they are crucial indicators that shape future educational and career opportunities, especially in college admissions (Erbes et al., 2021; Geiser and Santelices, 2007). Grades reflect not only students’ academic knowledge but also their effort, participation, and behavior, making them a multidimensional assessment tool (Bowers, 2019). For high school students, grades are reliable predictors of college success, correlating strongly with both freshman year performance and long-term academic outcomes in higher education (Galla et al., 2019; Hodara and Lewis, 2017). This suggests that grades reflect a student’s comprehension and skill acquisition, significantly impacting their chances of university admission and future academic success.

Academic grades are especially important for Chinese high school students as well, where academic grades carry immense weight due to the highly competitive nature of the national college entrance examination system, commonly known as the *gaokao* (Gao et al., 2023). In China, the *gaokao* is not just an examination; it is determining access to high quality universities and future career prospects (Liu and Helwig, 2022). The pressure to achieve high academic grades in China is intense (Gao et al., 2023), success in the *gaokao* can open doors to elite institutions and highly sought-after career paths, while lower grades can significantly limit a student’s options, especially for rural area students (Chen, 2022). For these students, high grades in the *gaokao* are one of the few viable paths to entering universities and improving their socioeconomic status.

Previous studies have consistently shown that positive Teacher–Student Relationships (TSRs) are significantly related to students’ academic grades (Robinson et al., 2022). TSRs are related to students’ autonomous motivation and learning experiences, which, in turn, positively influence their academic performance (Robinson et al., 2022; Maulana et al., 2014). Moreover, TSRs play a crucial role in fostering students’ socio-emotional development, which enhances their emotional well-being and resilience, further supporting their academic engagement (Jowett et al., 2023). Positive TSRs are associated with improved classroom engagement and learning outcomes (Baafi, 2020). Positive relationships with teachers create an environment where students feel valued and supported, leading to increased effort, participation, and persistence in their academic tasks (Baafi, 2020). Such relationships not only provide academic guidance but also offer emotional support, helping students to manage stress and overcome challenges that may arise during their educational journey (Vos et al., 2020; Dietrich et al., 2020).

Despite the established positive relationship between TSRs and academic grades, previous studies have largely focused on general student populations, often overlooking the unique challenges faced by rural Chinese students, who experience high levels of academic pressure and limited access to mental health resources (Zee and Koomen, 2016). Additionally, while much research has documented the direct effects of TSRs on academic outcomes, fewer studies have examined the moderating roles of mental health symptoms and academic resilience within this context. Little is known about how these individual factors shape the effectiveness of TSRs in promoting academic success among students facing heightened psychological challenges or varying levels of resilience. By exploring these moderating effects, the current study addresses this gap, providing a more comprehensive understanding of the factors influencing academic success in rural Chinese students.

## 2 Theoretical framework and hypotheses development

### 2.1 Teacher–student relationships and academic grades

TSRs play a pivotal role in promoting academic success. Students rely on the guidance and support of their teachers to develop essential academic skills, set educational goals, and make informed decisions about their learning journey (Wentzel et al., 2016; Midgley et al., 2015). Teachers’ encouragement helps students overcome challenges, build resilience, and foster a positive academic identity (Wentzel et al., 2016). Positive TSRs are associated with improved academic achievement, higher levels of motivation, better attendance, and reduced behavioral issues and dropout rates (Roorda et al., 2021; Beck, 2019). These relationships create a favorable learning environment that enhances both students’ social and academic development, leading to increased student engagement and academic performance. TSRs in schools act as a promoting, safeguarding, or risk element for students in developing the essential skills and resources required for their success and well-being in school (Ansari et al., 2020).

Attachment theory provides a strong theoretical foundation for understanding the role of TSRs in student development (Bowlby, 1979). The theory posits that adult-child relationships, including those with teachers, serve as a secure base for students, enabling them to explore their learning environments and develop cognitive and self-regulatory skills (Ainsworth et al., 2015). Students who form positive attachments with their teachers are more likely to engage in learning, collaborate with teachers, and persist in mastering challenging tasks (Pianta, 1999). On the other hand, students who lack a feeling of security in their relationship with a teacher may become withdrawn and disengaged from learning (Martin and Collie, 2019), or they might exhibit emotional or behavioral conflicts with the teacher (Baker et al., 2008). Positive TSRs supporting students’ academic success and emotional well-being (Bull et al., 2020).

In rural areas of China, the influence of TSRs on students is particularly significant. Many students in these regions study and live at school, especially in rural high schools where boarding is common (Shi and Sercombe, 2020). Between 2001 and 2012, the Chinese central government implemented several policies to redistribute educational resources in response to changes in administration and finance within the national education system (Shi and Sercombe, 2020). Notably, the School Consolidation Policy (*che dian bing xiao*) aimed to merge smaller rural schools into larger ones. For those students from rural area, who often spend extended periods away from their families, TSRs become even more crucial, serving not only as an academic support system but also as an emotional anchor. The time spent at school and the lack of immediate family presence mean that positive TSRs play a far more critical role in rural student’s overall development and well-being compared to urban students, who may have more access to external support networks (Yang et al., 2023). Understanding the unique dynamics of TSRs in these contexts is crucial for addressing the academic and social challenges faced by rural students. Given the importance of TSRs in promoting academic success, particularly in the Chinese context, the following hypothesis is proposed:

*Hypothesis 1*: TSRs are significantly associated with students’ academic grades.

### 2.2 The moderating role of mental health symptoms on the relationship between TSRs and academic grades

Mental health is a fundamental component of overall well-being, enabling individuals to manage stress, reach their potential, work productively, and contribute to their community (World Health Organization, 2022). While mental health refers to a state of optimal functioning, mental illness involves diagnosable conditions that impair thinking, emotional regulation, or behavior (American Psychological Association, 2021). These conditions range from common disorders like anxiety and depression to more severe disorders such as schizophrenia and bipolar disorder, which can significantly disrupt daily functioning (World Health Organization, 2022). Many individuals, particularly adolescents, experience psychological distress that, while not meeting the clinical criteria for a formal diagnosis, can still significantly impact well-being and daily functioning (World Health Organization, 2022). These subclinical symptoms are crucial for understanding the broader spectrum of mental health challenges, as they can often go unnoticed but still affect individuals’ quality of life (National Institute of Mental Health, 2022). To capture this spectrum, this study adopts the term “mental health symptoms,” which aligns with the World Health Organization’s International Classification of Diseases, Eleventh Revision (ICD-11), emphasizing that symptoms vary in severity and do not always meet the criteria for a formal diagnosis (Harrison et al., 2021).

Students experiencing high levels of anxiety or depressive symptoms often struggle with concentration, memory, and overall cognitive functioning, which are essential for learning and academic success (Eisenberg et al., 2009). Cognitive Theory (Sweller, 1988) and Attentional Control Theory (Eysenck et al., 2007) suggest that mental health symptoms place additional cognitive demands on students, reducing the mental resources available for learning. Students experiencing anxiety, depression, or high levels of stress might find it harder to engage academically, even in the presence of strong TSRs, as their mental health challenges may limit their ability to focus, participate, and stay motivated (Burić and Kim, 2020). The emotional and academic support provided by teachers might not fully counterbalance the adverse effects of poor mental health on learning outcomes (Roorda et al., 2021). Therefore, mental health symptoms may act as a moderator in the relationship between TSRs and academic grades. The strength of the positive association between TSRs and academic grades may weaken for students experiencing higher levels of anxiety, depression, or stress. Although positive TSRs can contribute to academic success, the cognitive and emotional burdens imposed by mental health issues might prevent students from fully benefiting from these relationships. Therefore, it is hypothesized that:

*Hypothesis 2*: Mental health symptoms moderate the relationship between TSRs and academic grades.

### 2.3 The conditional moderating role of mental health symptoms and academic resilience in the TSRs-academic grades

Academic resilience is the ability to overcome challenges and persist in academic tasks, even under difficult circumstances (Radhamani and Kalaivani, 2021). This capacity is not fixed but is shaped by various factors, including personal characteristics, school environment, and broader societal influences (García Crespo et al., 2022). Resilience Theory highlights that resilience involves interacting with both risk factors—such as mental health symptoms—and protective factors, including supportive relationships and coping strategies (Masten et al., 2021). Within this framework, resilience acts as a buffer, enabling students to adapt positively despite adversity.

In education, academic resilience helps students manage the cognitive and emotional burdens associated with mental health symptoms, such as anxiety, depression, and stress, allowing them to maintain academic performance (Rutter, 2012). For instance, students with high levels of resilience may utilize adaptive coping strategies to deal with academic pressure and emotional distress (Masten, 2014). These strategies can include seeking social support, problem-solving, and reframing negative thoughts, all of which can help mitigate the impact of mental health symptoms on cognitive processes essential for learning (Masten, 2014). Moreover, academic resilience is particularly significant for students from disadvantaged backgrounds (Agasisti et al., 2018). Socioeconomically disadvantaged students, such as those from rural areas, are more likely to exhibit lower academic outcomes due to a range of factors, including limited access to resources, higher stress levels, and lower support systems (Buchmann, 2002; Sirin, 2005). Resilience can explain why some students, despite facing socio-economic disadvantages, manage to achieve educational success (Masten, 2014).

Academic resilience characterizes students who succeed in school despite the presence of adversity, enabling them to cope with the same risks that others may find overwhelming (Sandoval-Hernández and Białowolski, 2016). For example, resilience might allow students to effectively moderate the negative impact of mental health symptoms on their academic performance by utilizing available TSRs as a support mechanism. While mental health symptoms can weaken the positive effects of TSRs by reducing students’ ability to engage fully in classroom activities, academic resilience can act as a conditional moderator in this relationship. Students with high academic resilience are better equipped to leverage TSRs as a source of support, even when struggling with mental health issues. Conversely, students with low resilience may struggle to benefit from positive TSRs in the presence of mental health symptoms, leading to poorer academic grades. In this study, we hypothesize that academic resilience moderates the relationship between mental health symptoms and the TSRs-academic grades relationship.

*Hypothesis 3*: Academic resilience serves as a conditional moderator in the relationship between mental health symptoms and the TSRs-academic grades relationship.

### 2.4 Current study

**Figure 1:**
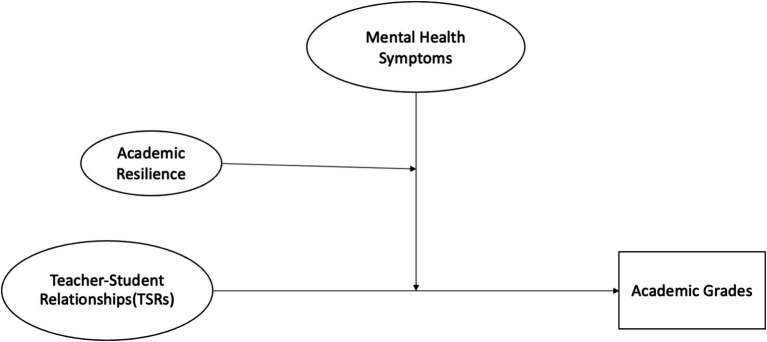
Research framework.

This study aims to address a critical gap in understanding how varying levels of academic resilience and mental health symptoms conditionally moderate the effectiveness of TSRs in fostering academic success. Figure 1 provides the research framework of this study.

Figure 1 illustrates how mental health symptoms are hypothesized to moderate the relationship between TSRs and academic grades, potentially weakening the positive effects of TSRs on academic performance. Academic resilience, in turn, is expected to serve as a conditional moderator, further moderating the impact of mental health symptoms on the TSRs-academic grades relationship. This means that students with higher resilience are better positioned to overcome the cognitive and emotional burdens of mental health symptoms, thereby continuing to benefit from positive TSRs. Conversely, students with lower resilience may struggle to leverage TSRs effectively when faced with psychological challenges, leading to poorer academic outcomes. By focusing on the conditional moderating role of academic resilience, this research aims to provide deeper insights into the mechanisms through which resilience and mental health symptoms influence academic outcomes.

## 3 Methodology

### 3.1 Sample size and participants

The sample size for this study was calculated according to SEM guidelines, considering the number of latent variables and their corresponding indicators (Hair et al., 2014). Table 1 summarizes the minimal sample sizes necessary for various configurations of latent variables and indicators within SEM analysis.

**Table 1:** Minimal sample size in SEM analysis.

Sample size required	Criteria from the research model
100 participants	Consists of no more than five latent variables, each with a minimum of three indicators.
150 participants	Contains no more than seven latent variables, each of which has three or more indications.
300 participants	Includes no more than seven latent variables, each with no more than three indicators.
500 participants	More than seven latent variables, each with fewer than three indicators, make up this variable.

Given that the model in this study includes three latent variables, 500 questionnaires were distributed to high school students in China. Of these, 433 students responded.

### 3.2 Demographic controls

Demographic conditions were meticulously controlled in this study. Demographic variables controlled for included age, household income, geographical background, and family structure. Family income was categorized as: below 10,000 annually, between 10,000 and 50,000, and above 50,000. Additionally, geographical background was coded as 1 for rural and 2 for urban settings. Family structure was categorized as: living with both biological parents, living with one parent and one stepparent, and other family structures. The socio-demographic background of the study participants, along with these classifications, is detailed in Table 2.

**Table 2:** The socio-demographic background of study participants (*n* = 433).

Category	Subcategory	Details
Participant demographics	Total participants	433 teenagers
Age	Below 16 years old	0
	Within 16–18 years old	387 (90%)
	Over 18 years old	46 (10%)
Geographical background	Rural	407 (96%)
	Urban	26 (4%)
Family income (yuan)	Below 10,000	175 (40.4%)
	Between 10,000 and 50,000	212 (48.9%)
	Above 50,000	46 (10%)
Family structure	Living with both biological parents	365 (84.2%)
	Living with one parent and one stepparent	43 (9.9%)
	Other	25 (5.7%)

Following the application of filters based on these demographic controls—participants above 16 years old, from rural areas, family income below 50,000 yuan, and those living with both biological parents—337 datasets met the criteria for this study.

### 3.3 Procedures

The study received approval from the Institutional Ethical Committee (IEC) (Approval Code: IEC-2024-FOSSLA-0012). Informed consent was not obtained directly from the students. The permission was granted by their parents and teachers, who were thoroughly briefed on the study’s objectives, procedures, and the voluntary nature of participation. Students were also informed about the study in an age-appropriate manner and clearly notified of their right to withdraw at any time without consequences, ensuring student autonomy. Before the main data collection, a pre-test phase was conducted to refine the questionnaire for clarity and relevance. Data collection assistants, primarily high school teachers, received training to ensure consistency and accuracy in administering the survey. Based on feedback from the pre-test, adjustments were made, and the final version of the questionnaire was distributed online by the research assistants. Participation was entirely voluntary, and students were made aware of the availability of counseling services in case they experienced any discomfort following the survey.

### 3.4 Measures

#### 3.4.1 Teacher–student relationships scale

This study obtained permission and adapted the ‘Teacher–Student Relationship Scale’ from the ED School Climate Surveys STUDENT SURVEY, developed by U.S. Department of Education, National Center for Education Statistics (n.d.). The scale was adapted to ensure cultural relevance and alignment with the specific educational context of rural Chinese students. Items were modified to better reflect the local learning environment and make the questions more relatable to the target population. To test the effectiveness of the adapted scale and ensure its reliability and validity, a pilot study was conducted prior to the main data collection. This adaptation and testing process ensured that the scale maintained its reliability and validity while being applicable to the unique setting of the study.

Recognizing that students have varied subjective experiences with different teachers, this study focuses specifically on mathematics TSRs to more accurately capture the dynamics between students and teachers. Mathematics was chosen because of its central role as a core subject in academic achievement, particularly in the Chinese educational system. As a high-stakes subject with standardized assessments, mathematics often has significant implications for students’ future educational and career opportunities. Consequently, the relationship between students and their mathematics teacher is critical in shaping academic success. Given the importance of mathematics grades in overall academic performance evaluation, examining TSRs in this subject provides valuable insights into how these relationships influence academic outcomes. Sample items included “My mathematics teacher cares about me,” “I have a good relationship with my mathematics teacher,” and “I like my mathematics teacher.” The items were rated on a 5-point scale from 1 (completely disagree) to 5 (completely agree). The fit indices from a confirmatory factor analysis (CFA) were [*χ*^2^/df = 2.798, CFI = 0.998, TLI = 0.994, RMSEA (90% CI) = 0.036, SRMR = 0.005].

#### 3.4.2 Academic grades

This study calculates the average of three recent test grades for each student to derive a comprehensive measure of academic grades in mathematics. This average serves as a quantifiable indicator of their academic grades in the subject. By focusing on recent test scores, the study ensures that the data reflects students’ current understanding and mastery of mathematical concepts, providing a more accurate and timely assessment of their academic achievements. This approach aligns with the measure of TSRs, which specifically examines the relationship between students and their mathematics teacher. This alignment enables a direct exploration of how the quality of the teacher–student relationship in mathematics related with students’ academic performance in this key subject.

#### 3.4.3 Academic resilience scale

To measure academic resilience, this study adapted several items from the Connor-Davidson Resilience Scale (CD-RISC) (Connor and Davidson, 2003). The selected items were specifically modified to focus on the academic context. These adaptations ensure that the scale evaluates how students manage academic stressors, persist through difficulties, and maintain a growth mindset. Sample items include “I can adapt to changes in academic expectations and circumstances with relative ease.” “Even when faced with significant academic difficulties, I persist and do not give up easily.” “I often see academic challenges as opportunities for growth rather than insurmountable problems.” Respondents rated each statement on a 5-point scale, where 1 represents “not true at all” and 5 means “true all the time.” The fit indices from CFA were [*χ*^2^/df = 1.289, CFI = 0.999, TLI = 0.996, RMSEA = 0.028, SRMR = 0.012].

#### 3.4.4 Mental health problems scale

In this study, the Depression, Anxiety, and Stress Scale (DASS-21) was utilized and adapted to assess the mental health symptoms of students (Lovibond and Lovibond, 1995). The selected items reflect the key emotional states we aimed to measure. For example, item 1 addresses the absence of positive affect, item 3 focuses on physical symptoms of anxiety, and item 11 pertains to anhedonia (lack of interest or feeling). The fit indices from CFA were [*χ*^2^/df = 1.854, CFI = 0.986, TLI = 0.979, RMSEA (90% CI) = 0.052, SRMR = 0.024].

### 3.5 Data analyses

Mplus 8.3 software was employed for the analyses in this study. In case of missing data, maximum likelihood (ML) estimation was used within the Structural Equation Modeling (Muthén and Muthén, 2017). The widespread acceptance of ML in academic research is attributed to its validated properties (Kline, 2023). Additionally, the study applied the Maximum Likelihood with Robust standard errors (MLR) method utilized for conditional moderation analysis. MLR computes standard errors using the sandwich estimator technique and aligns the chi-square test statistic closely with the Yuan-Bentler T2* test statistic (Bollen, 1989). The model fit was evaluated using several metrics, such as the ratio of chi-square value to degrees of freedom (*χ*^2^/df), the comparative fit index (CFI), the Tucker-Lewis index (TLI), root mean square error of approximation (RMSEA), and standardized root mean residual (SRMR). Typically, *χ*^2^/df of less than 5, CFI and TLI values equal to or greater than 0.90, and RMSEA and SRMR values of 0.08 or lower indicate an acceptable model fit (Marsh et al., 2004; Baron and Kenny, 1986). This comprehensive approach enabled a detailed examination of the direct, indirect, mediating, and moderating effects within the relationships under study.

## 4 Results

### 4.1 Reliability and validity

#### 4.1.1 Measurement model

Before proceeding with hypothesis testing in the structural model, it is essential to confirm the constructs’ internal consistency reliability, convergent validity, and discriminant validity (Anderson and Gerbing, 1988). This step is crucial in SEM, as it provides a foundation for credible hypothesis testing. Additionally, this step refines the model by excluding certain items through CFA. The refinement process is based on parameters such as factor loadings (estimates), standard errors (S.E.), *Z*-scores, *p*-values, and R-squared values (Kline, 2023). These parameters help assess the strength and significance of the relationships between observed variables and their respective latent constructs. The reliability results are presented in Table 3.

**Table 3:** Reliability and convergent validity scale.

Dim	Item	Parameters of significance test				Item reliability	Composite reliability	Convergence validity
		Estimate	S.E.	*Z*	*p* Value	*R* square	CR	AVE
TSRs	SR2	0.721	0.038	18.999	***	0.520	0.796	0.566
	SR4	0.802	0.036	22.337	***	0.643		
	SR5	0.732	0.038	19.475	***	0.536		
MHS	M1	0.724	0.037	19.661	***	0.524	0.750	0.500
	M3	0.701	0.038	18.355	***	0.491		
	M5	0.697	0.038	18.161	***	0.486		
	M11	0.721	0.037	19.530	***	0.520		
AR	R1	0.760	0.033	23.228	***	0.577	0.826	0.543
	R8	0.735	0.034	21.560	***	0.540		
	R10	0.756	0.033	23.113	***	0.573		
	R13	0.693	0.037	18.908	***	0.480		

TSRs, Teacher–Students Relationships; MHS, Mental Health Symptoms; AR, Academic Resilience. ***p < 0.001.

Table 3 presents the reliability results. According to Hair et al. (2019), ensuring that individual items are reliable indicators of their respective dimensions is crucial. In this study, all dimensions show Composite Reliability (CR) values exceeding 0.7, indicating strong internal consistency and reliability for each scale. High CR values demonstrate that the items within each variable are cohesive and effectively measure the intended construct (Hair et al., 2019). Moreover, all variables report Average Variance Extracted (AVE) values greater than 0.5, indicating that more than 50% of the variance is attributable to the construct itself rather than measurement error, thus supporting solid convergent validity (Fornell and Larcker, 1981). The high *Z* values, coupled with significant *p*-values (*p* < 0.001), further confirm the statistical significance of each item’s factor loadings onto their corresponding dimensions, reinforcing the robustness of the measurement model.

To further understand the constructs within this study, we conducted an in-depth analysis focusing on convergent calibration and differential calibration. Convergent calibration assesses whether different items measuring the same construct agree, which is essential for establishing the reliability and validity of the constructs. Differential calibration examines the extent to which these constructs differ from one another, thereby establishing the discriminant validity of the measurement model. Table 4 presents the results of these analyses.

**Table 4:** Convergent calibration and differential calibration analysis table.

Dim	Item reliability	Composite reliability	Convergence validity	Discriminate validity		
	ST. Loading	CR	AVE	TSR	TPQ	MHI
TSRs	0.721–0.802	0.796	0.566	0.752		
AR	0.693–0.760	0.826	0.543	0.313	0.737	
MHS	0.697–0.724	0.750	0.500	0.266	0.409	0.707

TSRs, Teacher–Student Relationships; AR, Academic Resilience; MHS, Mental Health Symptoms.

Table 4 summarizes the discriminant validity. Discriminant validity was confirmed by comparing the square roots of AVEs to inter-construct correlations (Fornell and Larcker, 1981). In all cases, the square roots of AVEs for TSRs (0.752), AR (0.737), and MHS (0.707) were greater than their respective correlations with other constructs, demonstrating that each construct is distinct from the others. This highlights the good discriminant validity of the model. Therefore, the analysis indicates that all constructs in the model meet the criteria for internal consistency, convergent validity, and discriminant validity, supporting the robustness of the measurement model.

#### 4.1.2 Structural model

After confirming the reliability and validity of the measurement model, the next critical step in SEM analysis is assessing the structural model (Gerbing and Anderson, 1988). This step is essential for testing the hypothesized relationships between constructs and evaluating the explanatory power of the theoretical model. In the initial application of the Latent Moderated Structural (LMS) method using Mplus, conventional fit indices such as chi-square, TLI, and CFI are not provided. These indices rely on normality assumptions, which do not apply to the LMS approach. To prevent misinterpretation, Mplus developers have intentionally excluded these indices from the LMS output. Instead, model evaluation relies on information criteria indices, such as the Akaike Information Criterion (AIC) and Bayesian Information Criterion (BIC) (Yang, 2005).

One approach to evaluating model fit is by first analyzing a baseline model that includes the moderators and their direct effects on the dependent variable, without incorporating any latent interaction terms (Sardeshmukh and Vandenberg, 2017). This initial model is assessed using Maximum Likelihood (ML) estimation. Once the baseline model is evaluated, a more complex model that includes latent interaction terms is developed and compared against the baseline model using information criteria like AIC and BIC. A well-fitting baseline model is preferable before adding interaction terms. If the baseline model has a poor fit, adding interactions may be ineffective. Conversely, if the baseline model fits well, introducing interaction terms allows for assessing how they affect the information criteria (Burnham and Anderson, 2002). The baseline model fit indices are presented in Table 5.

**Table 5:** Baseline model fit indices.

Index	Criteria	Baseline model
CH-SQR	SMALLER IS BETTER	115.783
DF	LARGER IS BETTER	52
CHI-SQR/DF (*χ*^2^/df)	3 > NORM CHI-SQR > 1	2.226
CFI	>0.90	0.948
TLI	>0.90	0.934
RMSEA	<0.08	0.062
SRMR	<0.08	0.059

CH-SQR, Chi-Square; DF, Degrees of Freedom; CFI, Comparative Fit Index; TLI, Tucker-Lewis Index; RMSEA, Root Mean Square Error of Approximation; SRMR, Standardized Root Mean Square Residual.

The Chi-Square statistic is a widely used measure of model fit, but it is highly sensitive to sample size, particularly in models with large samples (Kline, 2023). As the sample size increases, which may falsely suggest poor model fit. This sensitivity limits the reliability of using the Chi-Square statistic alone to evaluate model fit in large samples. To mitigate this issue, researchers often use the Chi-Square to Degrees of Freedom (*χ*^2^/df) ratio, which adjusts the Chi-Square value based on the model’s complexity, represented by the degrees of freedom. By dividing the Chi-Square statistic by the degrees of freedom, this ratio provides a more balanced and interpretable measure of model fit (Kline, 2023). A lower ratio (typically between 1 and 3) indicates that the model fits the data reasonably well, accounting for both sample size and model complexity. Additionally, CFI and TLI should be 0.90 or higher to indicate a good fit. RMSEA and SRMR should be 0.08 or lower for a good fit. Therefore, this baseline model exhibited a satisfactory fit [*χ*^2^/df = 2.2262, CFI = 0.948, TLI = 0.934, RMSEA = 0.062, SRMR = 0.059]. The baseline model fit indices meet all the usual criteria for judging a well-fitting model.

In practical applications, AIC is often favored over BIC for model comparisons (Burnham and Anderson, 2004; Yang, 2005). BIC tends to overly penalize models with more parameters due to certain unrealistic assumptions. AIC is considered to identify the model with the smallest mean square error, especially under the assumption of an infinite-dimensional true population model, which is not the case with BIC (Yang, 2005). This study selected AIC for model comparison because it is more effective at identifying the best-fitting model with less bias toward overly simple models, making it ideal for examining complex interaction effects. Similarly, the Adjusted BIC was chosen as it provides a more conservative fit index, which is adjusted to be more suitable for smaller sample sizes, thereby offering a balanced approach when assessing model complexity. The comparation results shown in Table 6.

**Table 6:** The comparation of baseline and interaction model fit indices.

Index	Criteria	Baseline model	Interaction model
AIC	LOWER IS BETTER	10,030.371	10,022.255
ADJUSTED BIC	LOWER IS BETTER	10,053.274	10,049.473

AIC, Akaike Information Criterion; BIC, Bayesian Information Criterion.

Table 6 indicated that the results reveal that the interaction model has a lower AIC value than the baseline model, suggesting greater parsimony while maintaining good fit. Similarly, the Adjusted BIC for the interaction model is also lower than that of the baseline model. Both the lower AIC and Adjusted BIC values for the interaction model indicate that it provides a better fit for the data than the baseline model. This improvement suggests that including the interaction term enhances the model’s explanatory power without introducing unnecessary complexity.

#### 4.1.3 Hypothesis testing

This study examines the relationship between TSRs and students’ academic grades, focusing on the moderating role of mental health symptoms and the conditional moderating effect of academic resilience. The first stage of analysis focuses on Hypothesis 1, which suggests that TSRs are significantly associated with students’ academic grades. Next, Hypothesis 2 tests the moderating effect of mental health symptoms on the relationship between TSRs and academic grades. Finally, Hypothesis 3 is evaluated, introducing academic resilience as a conditional moderator. This hypothesis posits that academic resilience further moderates the relationship between mental health symptoms and the TSRs-academic grades relationship. Table 7 presents the key findings of analysis, illustrating how mental health symptoms and academic resilience jointly moderate the relationship between TSRs and academic grades.

**Table 7:** Research model regression weight and hypothesis result in academic.

DV	IV	Estimate	S.E.	*Z*	*p*-Value	Hypothesis
ACADEMIC	TSRs	6.826	2.164	3.154	0.002	Support
	TSRs*MHS	−5.345	4.623	−1.156	0.248	Not support
	TSRs*MHS*AR	9.006	4.240	2.124	0.034	Support

TSRs, Teacher–Students Relationship; AR, Academic Resilience; MHS, Mental Health Symptoms.

Table 7 presents the results of the hypothesis testing. The coefficient for TSRs is positive (6.826) and statistically significant (*p* = 0.002), confirming a strong relationship between TSRs and academic grades. This supports the hypothesis that positive TSRs contribute to better academic achievement. However, the hypothesis suggesting that mental health symptoms would moderate the relationship between TSRs and academic grades was not supported. While the coefficient for the interaction between TSRs and mental health symptoms is negative (−5.345), indicating a potential weakening effect of mental health symptoms on the positive impact of TSRs, the effect is not statistically significant (*p* = 0.248). This suggests that in this study, students’ mental health symptoms did not significantly reduce the beneficial impact of TSRs on their academic outcomes. However, when academic resilience was introduced, it played a conditional moderating role in the interaction between TSRs and mental health symptoms. The positive coefficient (9.006) and a statistically significant *p*-value (*p* = 0.034) indicate that academic resilience might strengthens the positive effect of TSRs on academic performance, particularly when students experience higher levels of mental health symptoms. This result suggests that academic resilience might buffers the negative influence of mental health symptoms, allowing the positive relationship between TSRs and academic outcomes to remain strong. In other words, academic resilience helps maintain the benefits of positive TSRs, even in the presence of mental health challenges.

## 5 Discussion

This study presents a moderated moderation analysis involving TSRs, academic resilience, and mental health symptoms, showing that these factors collectively and significantly associated with students’ academic outcomes, such as academic grades. The positive coefficient for TSRs indicates that strong, supportive teacher–student relationships are fundamental to students’ academic success. This finding aligns with previous research, which consistently shows that positive TSRs are closely associated with higher levels of student engagement and academic achievement (Quin, 2017; Roorda et al., 2011). These relationships help foster a sense of belonging, motivation, and emotional security, all of which contribute to better academic outcomes. In contrast, the absence of significant moderating effects from mental health symptoms suggests that the relationship between TSRs and academic grades is complex and influenced by a variety of factors. Previous studies have emphasized the importance of mental health in academic success (e.g., Ansari et al., 2020), but the current findings indicate that mental health problems alone may not significantly moderate this relationship in the specific context of rural Chinese high school students. This result highlights the multifaceted nature of academic success and reinforces the idea that focusing on individual factors in isolation is insufficient. Instead, a holistic approach is needed to fully understand and support student achievement, considering both personal and contextual factors (Zhu et al., 2022).

The conditional moderating role of academic resilience suggests that personal factors such as resilience are crucial for academic success, particularly in the context of mental health challenges. Resilience appears to buffer the adverse effects of mental health symptoms on the relationship between TSRs and academic outcomes, indicating that students with higher levels of resilience may be better equipped to sustain their academic performance, even in the face of psychological distress. This finding aligns with research highlighting the protective role of resilience in educational contexts, where it serves as a key factor in helping students navigate challenges and persist in their studies (e.g., Martin and Marsh, 2009). Specifically, resilience might act as a buffer, reducing the negative impact of mental health symptoms on the positive influence of TSRs on academic grades. This underscores the importance of fostering both academic resilience and positive TSRs within educational environments. This emphasizes the need for educational policies and practices that address both emotional and academic needs, integrating resilience-building programs and promoting strong TSRs as part of a comprehensive approach to student well-being and achievement.

In rural Chinese communities, academic success is often viewed as the primary pathway to upward social mobility, leading to intense pressures, particularly as students prepare for the highly competitive national university entrance examination (*gaokao*). Within this context, academic resilience not only buffers students against the negative effects of mental health challenges but also embodies cultural values such as perseverance and dedication to academic excellence. These cultural expectations highlight the dual role of resilience: both as a coping mechanism for psychological distress and as a reflection of societal ideals regarding academic success. In conclusion, this study emphasizes the importance of holistic educational strategies that integrate emotional support, resilience-building, and positive teacher–student relationships. For students, particularly in high-pressure environments like rural China, resilience and TSRs play crucial roles in mitigating the negative effects of mental health problems on academic outcomes, ensuring that students are better equipped to succeed academically despite the challenges they face.

## 6 Conclusion

This study highlights the critical role of TSRs in promoting academic success among rural Chinese high school students. Strong, supportive TSRs were found to significantly enhance academic grades, confirming the importance of nurturing positive interactions between teachers and students. Although mental health problems alone did not significantly moderate this relationship, the combined interaction of academic resilience and mental health was significant. Students with higher resilience were better able to maintain academic performance despite psychological distress, underscoring the buffering role of resilience. Especially in high-pressure environments like rural China, fostering resilience and positive TSRs is vital to helping students cope with mental health challenges and achieve academic success. However, given the study’s focus on a specific educational context, future research should expand to other cultural and educational settings to enhance the generalizability of the results.

## Data Availability

The raw data supporting the conclusions of this article will be made available by the authors, without undue reservation.
